# Twelve–year (2008–2019) trends in socioeconomic inequalities in cardiovascular risk factors in a Swiss representative survey of the general population

**DOI:** 10.1016/j.pmedr.2024.102823

**Published:** 2024-07-14

**Authors:** Carlos de Mestral, Giovanni Piumatti, Mayssam Nehme, Idris Guessous, Silvia Stringhini

**Affiliations:** a Unit of Population Epidemiology, Division of Primary Care Medicine, Geneva University Hospitals, Geneva, Switzerland; b Fondazione Agnelli, Turin, Italy; c Department of Health and Community Medicine, Faculty of Medicine, University of Geneva, Geneva, Switzerland; d Division and Department of Primary Care Medicine, Geneva University Hospitals, Geneva, Switzerland; e University Center for General Medicine and Public Health, University of Lausanne, Lausanne, Switzerland

**Keywords:** Trends in socioeconomic inequalities, Relative inequalities, Absolute inequalities, Cardiovascular risk factors, Population-based study, Hypertension, Hypercholesterolemia, Diabetes, Obesity, Smoking, Sedentarity

## Abstract

**Objective:**

We assessed trends in socioeconomic inequalities in cardiovascular risk factors prevalence among Swiss adults from 2008 to 2019.

**Methods:**

Using data from the Bus Santé study, an annual survey of adults living in Geneva, Switzerland, we calculated the prevalence per period and by demographic and socioeconomic indicators, assessing inequality trends using the relative index of inequality (RII) and the slope index of inequality (SII).

**Results:**

Among 10,739 participants, most CVD risk factors decreased over time, while diabetes, obesity, and smoking prevalence remained steady. In 2017–2019, prevalence of most CVD risk factors was higher in socioeconomically disadvantaged groups. Relative and absolute inequalities decreased over time, but mostly remained, for hypertension [in 2017–2019, education-RII (95 % CI) = 1.27 (1.12–1.46), income-RII = 1.27 (1.10–1.47)], hypercholesterolemia [education-RII = 1.15 (1.00–1.32)], and sedentarity [education-RII = 1.95 (1.52–2.51), income-RII = 1.69 (1.28–2.23)], and appeared to have reversed for hazardous alcohol use [income-RII = 0.75 (0.60–0.93)]. Substantial and persistent relative and absolute inequalities in diabetes prevalence were observed [education-RII = 2.39 (1.75–3.27), income-RII = 3.18 (2.25–4.48), and subsidy-RII = 2.77 (1.89–4.05)]. Inequalities were also marked across all socioeconomic indicators for obesity prevalence [education-RII = 3.32 (2.63–4.19), income–RII = 2.37 (1.85–3.04), subsidy-RII = 1.98 (1.48–2.66)] and for smoking [education-RII = 2.42 (2.06–2.84), income-RII = 2.37 (1.99–2.84), subsidy-RII = 1.91 (1.56–2.35)].

**Conclusions:**

Over 12 years in Geneva, Switzerland, socioeconomic inequalities in hypertension, hypercholesterolemia, hazardous alcohol use, and sedentarity decreased but persist, while substantial inequalities in diabetes, obesity, and smoking remained unchanged.

## 1 Introduction

Cardiovascular disease (CVD) is a leading cause of death, morbidity, and disability worldwide, (Timmis et al., 2020) but the burden of disease disproportionally impacts socioeconomically disadvantaged populations. (Valero-Elizondo et al., 2018, Lopez and Adair, 2019, Abdalla et al., 2020, de Mestral and Stringhini, 2017) In high-income countries, despite favorable longitudinal trends in CVD, (Lopez and Adair, 2019) socioeconomic inequalities persist, (Valero-Elizondo et al., 2018, de Mestral and Stringhini, 2017, Powell-Wiley et al., 2022) which contribute to overburdening healthcare systems and hindering progress in reducing the CVD burden by 2025. Monitoring trends in socioeconomic inequalities in the prevalence of CVD risk factors in the population thus remains a top public health priority–it allows assessing the impact of existing public health and healthcare policies and strategies. (Girolamo et al., 2020) This is particularly important in the context of modifiable CVD risk factors, including hypertension, diabetes, hypercholesterolemia, smoking, alcohol misuse, increased body mass, and low physical activity, (Francula-Zaninovic and Nola, 2018) which are strongly associated with socioeconomic conditions, and which are, crucially, amenable to preventive and interventional strategies at both the population and healthcare levels. (Girolamo et al., 2020).

In Switzerland, CVD accounts for one third of all deaths and about 16 % of national health expenditures. (Gallino, 2017, Office, 2016) Although the overall prevalence of CVD risk factors and CVD incidence is generally low and mirrors that of other European countries with similar population demographics, (Timmis et al., 2020) previous studies have consistently found socioeconomic inequalities. (Stringhini et al., 2012, de Mestral et al., 2020, Marques-Vidal et al., 2011, Marques et al., 2018, Galobardes et al., 2003, Guessous et al., 2012, Faeh et al., 2011) For instance, in 2007, the national prevalence of obesity among individuals in the lowest tertile of household income was 10.0 % for men and 7.0 % for women, compared with 4.9 % and 3.0 %, respectively, among those in the highest income tertile. (Faeh et al., 2011) In 2017, the prevalence of diabetes among individuals with a monthly household income < 5000 CHF was 11.5 %, compared with 4.7 % among individuals with an income ≥ 9500 CHF. (de Mestral et al., 2020) Further, socioeconomically disadvantaged individuals are more likely to forgo healthcare, mainly due to costs, (de Mestral et al., 2022) even when receiving government health insurance subsidies. (Sandoval et al., 2021) Unsurprisingly, socioeconomically disadvantaged Swiss adults have been found to have between 1.2 and 2.3 greater odds of requiring hospitalization due to CVD than more socioeconomically privileged individuals. (Bayer-Oglesby et al., 2020) However, recent estimates and time trends in the prevalence of major CVD risk factors across different socioeconomic groups in Switzerland are currently lacking, while they are essential to assess, inform, and update public health preventive and interventional strategies.

Thus, using data from a population–based study in the Swiss state of Geneva over a 12–year period (2008–2019), we aimed to evaluate secular trends in socioeconomic inequalities in the prevalence of hypertension, diabetes, hypercholesterolemia, smoking, alcohol misuse, overweight, obesity, and sedentarity.

## 2 Methods

### 2.1 Sample

We used data from the Bus Santé study, a repeated cross–sectional population–based health survey in Geneva Canton, Switzerland. (de Mestral et al., 2020, Morabia et al., 1997) Every year since 1992, a stratified random sampling procedure according to age and sex distributions in the population of reference extracts a representative sample of the Canton’s noninstitutionalized residents from a local government residential list. Age range criteria were 35–74 years for annual surveys between 1992 and 2011 and 20–74 years thereafter. Unreachable contacts after three mailings and seven phone calls are discharged and replaced using the same extraction procedure described above. Mean participation rate was 48 % between 2008 and 2019 (Figure S1). Due to the COVID-19 pandemic, the Bus Santé study was paused after 2019. The Bus Santé study has been approved by the Ethics Committee of the Geneva University Hospitals, and all included participants signed written informed consent.

### 2.2 Data collection

Participants completed self–administered, standardized questionnaires covering a series of risk factors for major lifestyle–related chronic conditions, sociodemographic characteristics, educational and occupational histories, dietary intake, and physical activity. In addition, they were examined by health professionals in a temperature–controlled room. Health examinations took place in two clinics and one mobile medical unit. Blood pressure was measured thrice in the sitting position on the right arm after at least 10 min of rest using a validated automated oscillometric sphygmomanometer (OmronH HEM–907, Matsusaka, Japan). Body weight and height were measured using standard procedures and body mass index (BMI) was defined as weight / height (kg/m^2^). Fasting blood samples were collected and glucose, total and high–density lipoprotein (HDL), plasma cholesterol and triglycerides were assayed using commercially available enzymatic kits (Bayer Technicon Diagnostics, CV 1.4 %, 1.2 %, and 1.5 %, for glucose, cholesterol, and triglycerides, respectively).

### 2.3 Measures

As demographic variables we included sex (male, female), nationality (Swiss and non-Swiss), and age (categorized as < 50yrs and ≥ 50yrs and used in all descriptive and regression analyses as a binary variable). As socioeconomic indicators, we used the following variables: education, categorized as (1) primary/lower secondary, (2) higher secondary/apprenticeship, (3) tertiary; monthly household income in Swiss francs (1 CHF = 1.10 USD on 11 November 2023), (1) < 5000, (2) 5000–6999, (3) 7000–9499, (4) ≥ 9500 (available response options “I don’t know” and “I refuse to answer” were coded as missing); health insurance subsidy, (1) none, (2) partial, (3) full (available response option “I don’t know” was coded as missing). CVD risk factors included: (1) hypertension, defined as mean systolic and/or diastolic blood pressure ≥ 140/90 mmHg or self–reported hypertension or presence of anti–hypertensive medication; (2) diabetes, glycemia ≥ 7 mmol/L or self–reported diabetes or diabetes medication; (3) hypercholesterolemia, total blood cholesterol ≥ 6.5 mmol/L and high density lipoprotein < 1 mmol/L or self–reported hypercholesterolemia or cholesterol medication; (4) smoking (i.e. current smoker); (5) hazardous alcohol use, >10gr/day for women and > 20gr/day for men (based on recent national guidelines in Switzerland; Eidgenössische Kommission für Alkoholfragen, Orienterungshilfe zum Alkoholkonsum, 2018); (6) overweight, BMI (kg/m^2^) ≥ 25 but < 30; (7) obesity, BMI ≥ 30; and (8) sedentarity (based on a validated PAFQ questionnaire), (Richard et al., 2022) spending less than 10 % of daily activities in moderate- and high-intensity activities. (Varo et al., 2003).

### 2.4 Analyses

To increase statistical power, we grouped survey years into three periods: 2008–2010, 2011–2013, 2014–2016 and 2017–2019. We observed missing rates of 9 % for self–reported household income and 3 % for health insurance subsidy, which were due to the recoding of available response options “I don’t know” and “I refuse to answer” as missing. Using Little’s test to inspect missing values on educational level, household income and health insurance subsidy as a function of age category, sex and nationality, no statistically significant deviation from randomness was detected (*p = 0.06*). The same was observed when also including missing values for diabetes (0.1 %), smoking (0.1 %), alcohol use (2.5 %), BMI (1.2 %), and sedentarity (1.3 %) (*p = 0.36*).

Descriptive statistics are expressed as N (%) for categorical variables, and as median and interquartile range (IQR) for age. We calculated the prevalence (%, 95 % confidence intervals) of each CVD risk factor for the 2017–2019 period according to demographic and socioeconomic indicators using Poisson regressions with robust standard errors adjusting for age, sex, and survey year. (Barros and Hirakata, 2003) Then, to determine trends in socioeconomic inequalities we derived the relative index of inequality (RII) and the slope index of inequality (SII) for education, household income, and health insurance subsidy. (Mackenbach and Kunst, 1997) The RII and SII respectively describe the relative and absolute differences of the estimated rates between the two opposite extreme groups of the social hierarchy expressed on a continuous scale ranging from 0 (highest level) to 1 (lowest level). For example, a RII of 1.5 indicates a 50 % higher prevalence of an outcome in the most disadvantaged group compared with the most privileged group; similarly, a SII of 0.10 indicates that there are 10 more individuals with the outcome per 100 participants in the most disadvantaged group compared with the most privileged group. (Mackenbach and Kunst, 1997) Following established methods in the literature, we calculated the RII (95 % CI) using log-Poisson regression and the SII (95 % CI) using linear regression, in both cases using pooled data across all survey years and adjusting for age, sex and survey period. (Sandoval et al., 2018, Sandoval et al., 2019, Moreno-Betancur et al., 2015) P–values for linear trends in RII and SII were obtained by adding interaction terms between RII/SII and survey period. Statistical significance was considered for p < 0.05. All analyses were performed using Stata 17 (StataCorp, College Station, TX: StataCorp LP).

## 3 Results

### 3.1 Sample description

Our analytical sample comprised 10′739 participants with complete sociodemographic data (87.3 % from a total sample of 12′294; mean participation rate 48 %) (Table 1). Compared with included participants, the excluded group had a greater proportion of women, individuals aged < 50 years, and non-Swiss, and a lower proportion of tertiary educational level and higher income categories (Table S1). Among the included participants, median age (IQR) was 48 years (39–59), with 45.9 % of participants aged ≥ 50 years, and 51.0 % being women. Most held a secondary education degree or higher, reported a monthly household income of at least 7000 CHF, and received no health insurance subsidy (Table 1).

**Table 1:** Description of sample of adults residing in Geneva, Switzerland, Bus Santé 2008–2019.

	**Total**	**2008–2010**	**2011–2013**	**2014–2016**	**2017–2019**
**N**	10,739	2355	2556	2744	3084
**Age** in years, median (IQR)	48 (39–59)	50 (42–60)	48 (39–60)	47 (37–57)	47 (38–58)
**Age** groups					
<50yrs	5806 (54.1)	1133 (48.1)	1392 (54.5)	1557 (56.7)	1724 (55.9)
≥50yrs	4933 (45.9)	1222 (51.9)	1164 (45.5)	1187 (43.3)	1360 (44.1)
**Women**	5476 (51.0)	1207 (51.3)	1291 (50.5)	1386 (50.5)	1592 (51.6)
**Swiss nationality**	7236 (67.4)	1658 (70.4)	1747 (68.4)	1745 (63.6)	2086 (67.6)
**Educational level**					
Primary	3604 (33.6)	934 (39.7)	896 (35.1)	868 (31.6)	906 (29.4)
Secondary	2586 (24.1)	573 (24.3)	638 (25.0)	679 (24.7)	696 (22.6)
Tertiary	4549 (42.4)	848 (36.0)	1022 (40.0)	1197 (43.6)	1482 (48.1)
**Household income**, CHF/month					
<5000	2348 (21.9)	513 (21.8)	588 (23.0)	593 (21.6)	654 (21.2)
5000–6999	1971 (18.4)	430 (18.3)	492 (19.2)	515 (18.8)	534 (17.3)
7000–9499	2204 (20.5)	531 (22.5)	507 (19.8)	552 (20.1)	614 (19.9)
≥9500	4216 (39.3)	881 (37.4)	969 (37.9)	1084 (39.5)	1282 (41.6)
**Health insurance subsidy**					
None	9133 (85.0)	2021 (85.8)	2130 (83.3)	2339 (85.2)	2643 (85.7)
Partial	1197 (11.1)	243 (10.3)	313 (12.2)	325 (11.8)	316 (10.2)
Full	409 (3.8)	91 (3.9)	113 (4.4)	80 (2.9)	125 (4.1)

Note. Values are N (%) unless stated otherwise. IQR: interquartile range. Educational level: primary (primary/lower secondary), secondary (higher secondary/apprenticeship), tertiary. CHF: Swiss Francs (1 CHF = 1.10 USD on 11 November 2023).

### 3.2 Socioeconomic inequalities in CVD risk factors in 2017–2019

Table 2 presents the age– and sex–adjusted prevalence rates of CVD risk factors from the 2017–2019 survey period according to demographic and socioeconomic indicators. Participants with primary/lower education consistently exhibited higher prevalence of all examined risk factors compared to those with higher educational level, except for hazardous alcohol use and for sedentarity, which showed comparable rates across educational groups. For instance, hypertension prevalence was 29.1 % among those with primary/lower secondary education versus 22.7 % among those with a tertiary degree; similarly, hypercholesterolemia prevalence was 28.3 % versus 23.4 % for hypercholesterolemia, overweight prevalence was 36.3 % versus 27.3 %, obesity prevalence was 16.8 % versus 7.8 %, diabetes prevalence was 8.5 % versus 4.7 %, smoking prevalence was 26.9 % versus 15.2 %, and sedentarity prevalence was 15.4 % versus 8.3 %. Differences in CVD risk factors prevalence across household income groups followed a similar pattern, although hypercholesterolemia prevalence did not vary significantly by income level. Additionally, participants receiving a partial or full health insurance subsidy generally showed higher prevalence of CVD risk factors compared with those without any subsidy (Table 2).

**Table 2:** Prevalence of cardiovascular risk factors in adults residing in Geneva, Switzerland, Bus Santé 2017–2019.

	**Hypertension**	**Hypercholesterolemia**	**Overweight**	**Obesity**	**Hazardous** **alcohol use**	**Diabetes**	**Smoking**	**Sedentarity**
**Overall**	25.0 (23.6, 26.3)	25.0 (23.6, 26.3)	31.3 (29.8, 32.8)	11.4 (10.3, 12.5)	19.6 (18.0, 21.2)	6.1 (5.4, 6.9)	20.1 (18.8, 21.4)	11.9 (10.6, 13.2)
**Age group**								
<50yrs	13.8 (12.4, 15.3)	15.5 (14.0, 17.1)	27.1 (25.2, 29.1)	8.7 (7.4, 9.9)	14.7 (12.8, 16.6)	2.5 (1.8, 3.2)	22.5 (20.7, 24.3)	10.9 (9.2, 12.5)
≥50yrs	40.2 (37.7, 42.7)	37.8 (35.4, 40.3)	36.9 (34.5, 39.3)	15.1 (13.3, 16.9)	26.3 (23.5, 29.0)	11.1 (9.5, 12.7)	16.7 (14.8, 18.6)	13.3 (11.2, 15.4)
*P–value*^a^	*<0.001*	*<0.001*	*<0.001*	*<0.001*	*<0.001*	*<0.001*	*<0.001*	*0.07*
**Sex**								
Women	21.7 (19.9, 23.5)	22.2 (20.4, 24.0)	23.3 (21.3, 25.3)	10.0 (8.6, 11.4)	19.5 (17.3, 21.7)	5.5 (4.5, 6.5)	17.5 (15.8, 19.2)	11.3 (9.6, 13.1)
Men	28.6 (26.5, 30.6)	28.0 (26.0, 30.1)	39.8 (37.5, 42.1)	12.9 (11.3, 14.4)	19.8 (17.4, 22.1)	6.8 (5.6, 8.0)	22.9 (20.9, 24.9)	12.5 (10.6, 14.4)
*P–value*^a^	*<0.001*	*<0.001*	*<0.001*	*0.01*	*0.88*	*0.10*	*<0.001*	*0.39*
**Educational level**								
Primary	29.1 (26.5, 31.6)	28.3 (25.7, 30.9)	36.3 (33.4, 39.2)	16.8 (14.5, 19.0)	19.3 (16.4, 22.2)	8.5 (6.9, 10.0)	26.9 (24.2, 29.6)	15.4 (12.8, 18.1)
Secondary	23.5 (20.8, 26.2)	23.3 (20.5, 26.0)	32.7 (29.5, 35.9)	11.2 (9.1, 13.4)	18.0 (14.8, 21.2)	5.7 (4.1, 7.2)	21.5 (18.7, 24.2)	14.1 (11.2, 17.0)
Tertiary	22.7 (20.7, 24.7)	23.4 (21.4, 25.4)	27.3 (25.1, 29.5)	7.8 (6.4, 9.1)	20.2 (17.8, 22.6)	4.7 (3.6, 5.7)	15.3 (13.6, 17.1)	8.3 (6.7, 10.0)
*P–value*^a^	*<0.001*	*0.005*	*<0.001*	*<0.001*	*0.58*	*<0.001*	*<0.001*	*<0.001*
**Household income**, CHF/month								
<5000	29.9 (26.7, 33.0)	25.0 (21.9, 28.1)	32.2 (28.8, 35.7)	14.5 (11.8, 17.1)	18.8 (15.3, 22.4)	9.9 (7.8, 12.0)	28.5 (25.2, 31.9)	16.5 (13.1, 19.8)
5000–6999	26.6 (23.1, 30.1)	27.7 (24.1, 31.3)	35.5 (31.6, 39.5)	12.5 (9.7, 15.3)	21.6 (17.3, 25.8)	4.7 (3.0, 6.4)	21.3 (17.9, 24.7)	14.5 (10.9, 18.2)
7000–9499	27.0 (23.7, 30.2)	25.8 (22.5, 29.0)	33.9 (30.2, 37.5)	12.7 (10.1, 15.3)	17.3 (13.7, 20.9)	6.5 (4.7, 8.3)	19.7 (16.6, 22.8)	12.1 (9.0, 15.2)
≥9500	22.7 (20.5, 24.9)	25.3 (23.0, 27.6)	29.2 (26.7, 31.6)	8.7 (7.1, 10.2)	21.1 (18.4, 23.9)	4.8 (3.6, 6.0)	14.6 (12.7, 16.5)	7.6 (5.8, 9.3)
*P–value*^a^	*<0.001*	*0.84*	*0.06*	*<0.001*	*0.51*	*<0.001*	*<0.001*	*<0.001*
**Health insurance subsidy**								
None	24.8 (23.3, 26.3)	25.1 (23.6, 26.7)	31.3 (29.6, 33.0)	10.7 (9.6, 11.8)	20.6 (18.8, 22.4)	5.6 (4.8, 6.5)	18.8 (17.3, 20.2)	11.5 (10.1, 12.9)
Partial	26.4 (21.9, 30.9)	25.4 (20.8, 29.9)	33.2 (28.3, 38.1)	11.7 (8.2, 15.1)	15.4 (10.7, 20.0)	8.1 (5.1, 11.1)	24.4 (20.0, 28.8)	12.6 (8.4, 16.8)
Full	33.9 (26.6, 41.3)	30.8 (23.5, 38.1)	32.3 (24.5, 40.1)	21.2 (14.4, 28.1)	14.3 (6.8, 21.7)	13.0 (7.8, 18.2)	32.8 (24.9, 40.7)	10.4 (4.0, 16.8)
*P–value*^a^	*0.02*	*0.20*	*0.54*	*0.001*	*0.03*	*<0.001*	*<0.001*	*0.95*

Note. Values are percentages (95 % confidence intervals) unless stated otherwise. Prevalence and 95 % confidence intervals are from Poisson regressions with robust standard errors, adjusted for age, sex and survey year. CHF: Swiss Francs (1 CHF = 1.10 USD on 11 November 2023).

a P–value for difference between two groups and linear trend across three or more groups, based on F test.

Participants aged 50 years and older consistently exhibited higher prevalence of CVD risk factors compared to those aged 20–49 years, except for smoking, which was lower in the older group (16.7 % vs 22.5 %). Men had higher prevalence rates than women for hypertension (28.6 % vs 21.7 %), hypercholesterolemia (28.0 % vs 22.2 %), smoking (22.9 % vs 17.5 %), overweight (39.8 % vs 23.3 %), and obesity (12.9 % vs 10.0 %). Conversely, men and women had similar prevalence rates for diabetes (around 6.0 %), hazardous alcohol use (20.0 %), and sedentarity (around 12.0 %) (Table 2).

The age– and sex–adjusted prevalence of CVD risk factors during the previous survey periods are shown in Table S2 in the S upplement. The prevalence of most CVD risk factors decreased over time, while that of diabetes, obesity and smoking remained relatively constant. Table S3 shows the age- and sex-adjusted association of CVD risk factors prevalence with demographic and socioeconomic indicators across survey periods—results largely reflect the same pattern of CVD risk factors prevalence observed across demographic and socioeconomic groups.

### 3.3 Trends in socioeconomic inequalities in CVD risk factors prevalence

**Fig. 1:**
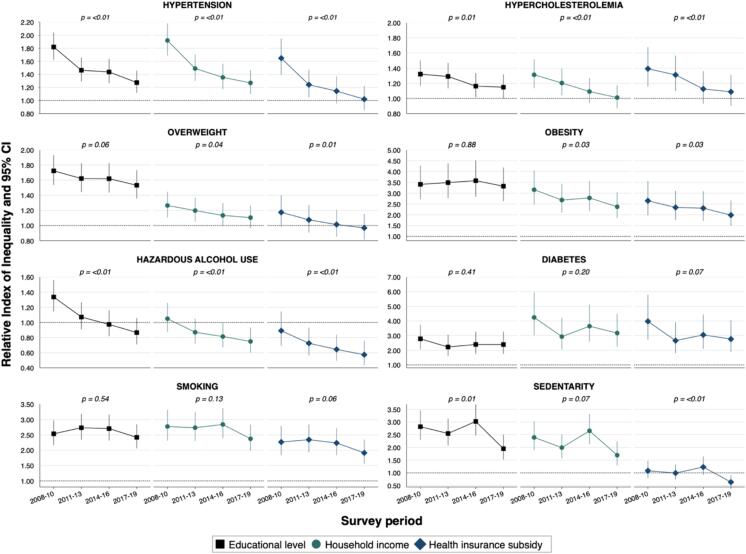
Trends in relative socioeconomic inequalities in the prevalence of cardiovascular risk factors among adults residing in Geneva, Switzerland, Bus Santé 2008–2019 Relative index of inequalities and 95 % confidence intervals are from Poisson regressions with robust standard errors, adjusted for age, sex and survey period. P values for linear trend are from interaction term between the RII and survey period. Dotted line RII = 1.00 indicates no difference in prevalence between most disadvantaged and most privileged socioeconomic groups.

**Fig. 2:**
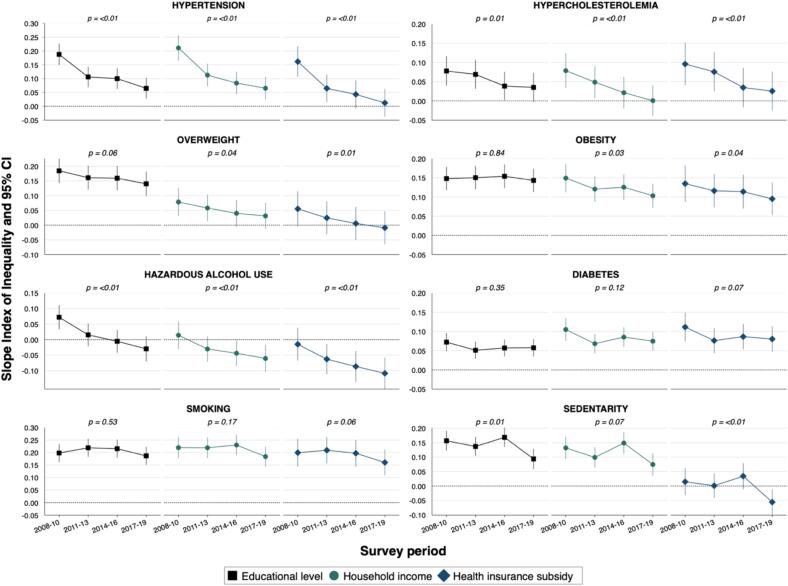
Trends in absolute socioeconomic inequalities in the prevalence of cardiovascular risk factors among adults residing in Geneva, Switzerland, Bus Santé 2008–2019 Slope index of inequalities and 95 % confidence intervals are from linear regressions with robust standard errors, adjusted for age, sex and survey period. P values for linear trend are from interaction term between the SII and survey period. Dotted line SII = 0.00 indicates no difference in prevalence between most disadvantaged and most privileged socioeconomic groups.

Fig. 1 depicts the trends in relative socioeconomic inequalities in CVD risk factors prevalence across survey periods, while Fig. 2 shows the trends in absolute socioeconomic inequalities (Tables S4 **and S5** in the **S upplement** provide detailed values).

Over the 12–year period, both relative and absolute inequalities in hypertension prevalence decreased significantly across all socioeconomic indicators (*linear trend p–value < 0.01* for all). For instance, the education-RII (95 % CI) decreased from 1.82 (1.62–2.04) in 2008–2010 to 1.27 (1.12–1.46) in 2017–2019 while the education-SII (95 % CI) decreased from 0.19 (0.15–0.23) to 0.06 (0.03–0.10). Similar trends were observed for hypercholesterolemia and overweight prevalence, for which relative and absolute inequalities generally decreased (Fig. 1, Fig. 2).

Both relative and absolute inequalities in obesity prevalence were marked for all socioeconomic indicators, and remained constant for educational level, with a RII of 3.32 (2.63–4.19) and a SII of 0.14 (0.11–0.17) during 2017–2019; in contrast, the RII and SII decreased for household income and health insurance subsidy (*p–trend = 0.03* for both). Concerning the prevalence of hazardous alcohol use, relative and absolute inequalities decreased overtime across all three socioeconomic indicators (*p–trend < 0.01*). For example, the education-RII went from 1.34 (1.14–1.56) to 0.87 (0.71–1.06) and the education-SII from 0.07 (0.03–0.11) to –0.03(–0.07–0.01). Both relative and absolute inequalities were marked and remained constant over time in the prevalence of diabetes according to all three socioeconomic indicators, with an income-RII of 3.18 (2.25–4.48) and an income-SII of 0.07 (0.05–0.10) during 2017–2019, for instance (Fig. 1, Fig. 2).

Similarly, relative and absolute inequalities in smoking prevalence remained constant over time; for example, in 2017–2019, the education-RII was 2.42 (2.06–2.84) and the education-SII was 0.19 (0.15–0.22). Concerning the prevalence of sedentarity, relative and absolute inequalities decreased over time across all socioeconomic indicators (Fig. 1, Fig. 2).

## 4 Discussion

In this study, we analyzed data from a large representative sample of adults residing in the canton of Geneva, Switzerland, to examine trends in socioeconomic inequalities in the prevalence of major modifiable CVD risk factors over a 12–year period (2008–2019). Our findings indicate that relative and absolute inequalities decreased over time in the prevalence of hypertension, hypercholesterolemia, overweight, and sedentarity, but remained substantial and unchanged for diabetes, smoking, and obesity. These persistent inequalities may contribute to the observed slow down and plateauing of the CVD mortality decline in high–income countries and underscore the need for both population–level and targeted public health interventions designed to meet the specific needs of socioeconomically disadvantaged groups. (Kivimäki et al., 2008, Bambra et al., 2010, Scholes et al., 2012, Mensah et al., 2018, Capewell and Graham, 2010).

Our results are consistent with previous studies on other Swiss populations (Stringhini et al., 2012, Marques-Vidal et al., 2011, Marques et al., 2018, Kaiser et al., 2012) and high–income countries. (Timmis et al., 2020, Abdalla et al., 2020, Schultz et al., 2018, Gaye et al., 2020, Truthmann et al., 2015, Kim et al., 2017, Dai et al., 2021) Lower educational and income levels were significant factors for higher prevalence of CVD risk factors, in line with prior research. (Abdalla et al., 2020, de Mestral and Stringhini, 2017, Odutayo et al., 2017, Rosengren et al., 2019) Sex differences in hypertension, hypercholesterolemia, smoking, overweight, and obesity also mirrored previous findings, (Stringhini et al., 2012, Appelman et al., 2015, Mosca et al., 2011) likely due to both biological and social processes. (Appelman et al., 2015, Bolijn et al., 2021, O’Neil et al., 2018) The reversal in inequalities in hazardous alcohol use prevalence may reflect a pattern of findings seen in several high–income countries where socioeconomically underprivileged individuals tend to abstain more from alcohol consumption than their more socioeconomically privileged counterparts. (Peña et al., 2017, Puddephatt et al., 2021).

The substantial and persistent socioeconomic inequalities in diabetes, obesity, and smoking prevalence mirror those found in other high-income countries. (Gaye et al., 2020, Hoebel et al., 2018, Odutayo et al., 2017, Rosengren et al., 2019) However, also in line with our findings, some countries have seen decreasing inequalities in certain CVD risk factors, such as the reduced hypertension inequalities reported Sweden between 1994 and 2014. (Eriksson et al., 2017) Socioeconomic inequalities in health outcomes are complex and influenced by a range of interrelated factors including socioeconomic conditions, access to healthcare, health behaviors, environmental factors, and psychosocial stressors. (Marmot, 2005) The decreasing inequalities in hypertension and hypercholesterolemia may be attributed to improved access to screening and management, (Gkiouleka et al., 2023, Schutte et al., 2023) increased awareness of cardiovascular health among disadvantaged populations, (Gkiouleka et al., 2023, Schutte et al., 2023) and a long term increase in physical activity within the Swiss population. (World Health Organization, 2020, Guessous et al., 2014).

Despite Switzerland’s high median income and generous welfare system, significant socioeconomic inequalities in CVD risk factors persist. The unchanged inequalities in diabetes likely indicate insufficient screening, management, and primordial, primary and secondary prevention efforts among socioeconomically disadvantaged groups. (Odutayo et al., 2017, Flatz et al., 2015, Gharacheh et al., 2024) Previous research in the Swiss population has shown higher rates of undiagnosed diabetes among disadvantaged individuals^12^ and a greater likelihood of renouncing healthcare for financial reasons, even when receiving health insurance subsidies. (de Mestral et al., 2022) Further, socioeconomically disadvantaged groups generally show higher prevalence of diabetes risk factors such as obesity5, (Powell-Wiley et al., 2022, Faeh et al., 2011) and sedentarity. (de Mestral and Stringhini, 2017, Powell-Wiley et al., 2022) This highlights the need for targeted public health efforts that address the specific barriers faced by socioeconomically underprivileged groups.

The persistence of socioeconomic inequalities in CVD risk factors presents a major obstacle for health systems in high–income countries as they work towards reducing the burden of CVD in their rapidly aging populations. (Lopez and Adair, 2019, Rosengren et al., 2019, Avan et al., 2019) Importantly, the COVID-19 pandemic, which disproportionally and adversely affected socioeconomically disadvantaged populations, may have erased progress or even worsened inequalities in CVD risk factors. (Laddu et al., 2023, Naylor-Wardle et al., 2021, Bann et al., 2023).

**Strengths and limitations**.

The main strength of this study is the eight CVD risk factors assessed across a 12–year period combining both self–reported answers and objective measures. In addition, to the best of our knowledge, this is the first study based on a Swiss adult population to simultaneously show prevalence trends across different major CVD risk factors and inequalities according to different sociodemographic and socioeconomic indicators. Limitations include the small sample size in the most socioeconomically disadvantaged groups, particularly that of participants receiving full health insurance subsidy, which may contribute to a lack of precision in the reported estimates. Moreover, due to the self–reported nature of the measures of household income and health insurance subsidy, we may have underestimated the real impact of socioeconomic conditions on CVD risk factors. Although partially accounted for by age stratification, age-cohort-period effects were not considered in our analyses, which may obscure cohort variations in socioeconomic inequalities. Finally, because the study was paused from March 2020 as a result of the COVID-19 pandemic, we were unable to assess the extent to which socioeconomic inequalities may have been impacted during and after the pandemic; this will be investigated as the yearly Bus Santé study resumed in 2023.

## 5 Conclusion

Using 2008–2019 population–based data from a Swiss adult population residing in Geneva, Switzerland, we found that relative and absolute socioeconomic inequalities decreased in the prevalence of hypertension, hypercholesterolemia, overweight, and sedentarity, though inequalities remain. Yet, persistent and substantial socioeconomic inequalities were found in the prevalence of diabetes, obesity, and smoking. These results highlight the need for tailored public health interventions addressing the needs of the most socioeconomically disadvantaged groups with the highest risk and burden of cardiovascular disease.

## CRediT authorship contribution statement

**Carlos de Mestral:** Writing – review & editing, Writing – original draft, Visualization, Methodology, Investigation, Formal analysis, Data curation. **Giovanni Piumatti:** Writing – review & editing, Writing – original draft, Visualization, Investigation, Formal analysis, Data curation. **Mayssam Nehme:** Writing – review & editing, Supervision, Project administration, Investigation, Conceptualization. **Idris Guessous:** Writing – review & editing, Validation, Supervision, Resources, Project administration, Methodology, Investigation, Funding acquisition, Formal analysis, Data curation, Conceptualization. **Silvia Stringhini:** Writing – review & editing, Validation, Supervision, Project administration, Methodology, Investigation, Funding acquisition, Formal analysis, Data curation.

## Declaration of competing interest

The authors declare that they have no known competing financial interests or personal relationships that could have appeared to influence the work reported in this paper.

## Data Availability

Data will be made available on request.
