# Supplementary material for: Twelve–year (2008–2019) trends in socioeconomic inequalities in cardiovascular risk factors in a Swiss representative survey of the general population
Source: Prev Med Rep. 2024 Jul 14;45:102823. doi: 10.1016/j.pmedr.2024.102823 (PMC11286997; doi:10.1016/j.pmedr.2024.102823)

**Supplementary material**

**Table S1.** Description of included and excluded participants, Bus Santé 2008-2019

**Table S2.** Trends in age- and sex-adjusted prevalence of cardiovascular risk factors according to demographic and socioeconomic factors, Bus Santé 2008–2019

**Table S3**. Trends in the age- and sex-adjusted association of CVD risk factors prevalence with demographic and socioeconomic indicators, Bus Santé 2008-2019

**Table S4.** Trends in relative socioeconomic inequalities in cardiovascular risk factors prevalence, Bus Santé 2008–2019

**Table S5.** Trends in absolute socioeconomic inequalities in cardiovascular risk factors prevalence, Bus Santé 2008–2019

**Figure S1.** Participation rate across the 2008–2019 period, Bus Santé

**Table S1**. Description of included and excluded participants

|  | **Included** | **Excluded** |
| --- | --- | --- |
| **N** | 10739 | 1559 |
| **Age** in years, median (IQR) | 48 (39-59) | 40 (25-54) |
| **Age** groups |  |  |
| <50 years | 5806 (54.1) | 1034 (66.5) |
| ≥50 years | 4933 (45.9) | 521 (33.5) |
| **Women** | 5476 (51.0) | 888 (57.1) |
| **Swiss nationality** | 7236 (67.4) | 935 (60.3) |
| **Educational level ^a^** |  |  |
| Primary | 3604 (33.6) | 469 (33.3) |
| Secondary | 2586 (24.1) | 455 (32.3) |
| Tertiary | 4549 (42.4) | 485 (34.4) |
| **Household** **income ^b^**, CHF/month |  |  |
| <5000 | 2348 (21.9) | 129 (31.5) |
| 5000–6999 | 1971 (18.4) | 62 (15.2) |
| 7000–9499 | 2204 (20.5) | 64 (15.6) |
| ≥9500 | 4216 (39.3) | 154 (37.7) |
| **Health insurance subsidy ^c^** |  |  |
| None | 9133 (85.0) | 945 (83.0) |
| Partial | 1197 (11.1) | 144 (12.7) |
| Full | 409 (3.8) | 49 (4.3) |
| **Hypertension** | 3158 (29.4) | 351 (22.5) |
| **Hypercholesterolemia** | 3020 (28.1) | 305 (19.6) |
| **Overweight** | 3496 (33.7) | 426 (29.0) |
| **Obesity** | 1278 (12.3) | 168 (11.4) |
| **Hazardous alcohol use** | 2090 (22.1) | 283 (20.5) |
| **Diabetes** | 739 (6.9) | 83 (5.3) |
| **Smoking** | 2272 (21.2) | 373 (24.0) |
| **Sedentarity** | 1406 (14.7) | 224 (16.1) |

Note. Values are N (%) unless stated otherwise. IQR: interquartile range. Educational level: primary (primary/lower secondary), secondary (higher secondary/apprenticeship), tertiary. CHF: Swiss Francs (1 CHF = 1.10 USD on 11 November 2023).

^a^ Education data were missing from 1.2% (N=149) of participants. Data shown for excluded participants with available household income and/or health insurance subsidy data.

^b^ Household income data were missing from 9.3% (N=1146) of participants. Data shown for excluded participants with available educational level and/or health insurance subsidy data.

^c^ Health insurance subsidy data were missing from 3.4% (N=417) of participants. Data shown for excluded participants with available educational income and/or household income data.

**Table S2.** Trends in prevalence of cardiovascular risk factors by demographic and socioeconomic factors, Bus Santé 2008–2019

|  | **Hypertension** |  |  |  |  | **Hypercholesterolemia** | | | |  |
| --- | --- | --- | --- | --- | --- | --- | --- | --- | --- | --- |
|  | 2008–2010 | 2011–2013 | 2014–2016 | 2017-2019 | *p-trend^a^* | 2008–2010 | 2011–2013 | 2014–2016 | 2017-2019 | *p-trend^a^* |
| **Overall** | 35.8 (34.0, 37.5) | 28.7 (27.2, 30.2) | 26.5 (25.0, 27.9) | 25.0 (23.6, 26.3) | *<0.01* | 31.0 (29.2, 32.7) | 28.1 (26.5, 29.6) | 25.2 (23.7, 26.6) | 25.0 (23.6, 26.3) | *<0.01* |
| **Age group**, y |  |  |  |  |  |  |  |  |  |  |
| ≥50 | 20.3 (18.0, 22.5) | 15.4 (13.6, 17.1) | 14.7 (13.1, 16.3) | 13.8 (12.3, 15.3) | *<0.01* | 19.9 (17.7, 22.1) | 16.5 (14.7, 18.3) | 15.5 (13.9, 17.1) | 15.5 (14.0, 17.1) | *0.03* |
| <50 | 49.7 (47.1, 52.3) | 45.8 (43.1, 48.5) | 43.6 (41.0, 46.3) | 40.2 (37.7, 42.7) | *<0.01* | 40.9 (38.3, 43.5) | 43.0 (40.3, 45.7) | 39.3 (36.6, 41.9) | 37.8 (35.4, 40.3) | *0.01* |
| **Sex** |  |  |  |  |  |  |  |  |  |  |
| Men | 41.3 (38.7, 43.8) | 32.7 (30.4, 35.0) | 31.9 (29.7, 34.1) | 28.6 (26.6, 30.6) | *<0.01* | 37.7 (35.1, 40.3) | 32.6 (30.3, 34.9) | 28.1 (26.0, 30.2) | 28.0 (25.9, 30.1) | *<0.01* |
| Women | 30.6 (28.2, 33.0) | 24.8 (22.8, 26.9) | 21.3 (19.4, 23.2) | 21.7 (19.9, 23.5) | *<0.01* | 24.6 (22.4, 26.9) | 23.7 (21.6, 25.8) | 22.4 (20.4, 24.3) | 22.2 (20.4, 24.0) | *0.74* |
| **Educational level** |  |  |  |  |  |  |  |  |  |  |
| Primary/lower secondary | 40.4 (37.6, 43.2) | 30.4 (27.8, 33.0) | 30.2 (27.6, 32.8) | 28.9 (26.4, 31.4) | *<0.01* | 33.0 (30.3, 35.8) | 29.5 (26.9, 32.1) | 27.3 (24.7, 29.8) | 28.2 (25.7, 30.7) | *0.18* |
| Secondary | 34.8 (31.1, 38.4) | 29.5 (26.3, 32.7) | 27.6 (24.7, 30.5) | 23.5 (20.7, 26.3) | *<0.01* | 28.0 (24.5, 31.5) | 27.4 (24.3, 30.6) | 24.0 (21.1, 26.9) | 23.3 (20.5, 26.1) | *0.19* |
| Tertiary | 30.3 (27.4, 33.2) | 26.7 (24.2, 29.2) | 22.4 (20.2, 24.5) | 22.7 (20.7, 24.7) | *0.01* | 30.2 (27.3, 33.1) | 27.2 (24.7, 29.8) | 24.1 (21.8, 26.3) | 23.4 (21.3, 25.4) | *0.01* |
| **Household income**, CHF/month |  |  |  |  |  |  |  |  |  |  |
| <5000 | 45.3 (41.4, 49.2) | 31.6 (28.3, 34.9) | 28.1 (24.7, 31.4) | 29.9 (26.7, 33.0) | *<0.01* | 36.9 (32.9, 40.9) | 30.1 (26.6, 33.5) | 26.8 (23.5, 30.1) | 25.0 (22.0, 28.0) | *<0.01* |
| 5000-6999 | 33.2 (29.0, 37.4) | 33.0 (29.2, 36.8) | 30.1 (26.5, 33.7) | 26.7 (23.1, 30.3) | *0.09* | 29.4 (25.3, 33.5) | 28.9 (25.1, 32.7) | 28.1 (24.5, 31.7) | 27.7 (24.1, 31.3) | *0.78* |
| 7000-9499 | 38.4 (34.5, 42.3) | 28.6 (24.9, 32.3) | 28.8 (25.2, 32.5) | 26.9 (23.7, 30.1) | *0.01* | 32.6 (28.8, 36.4) | 27.4 (23.8, 31.0) | 25.9 (22.4, 29.4) | 25.8 (22.5, 29.0) | *0.05* |
| ≥9500 | 28.6 (25.8, 31.4) | 26.2 (23.6, 28.8) | 25.9 (23.5, 28.3) | 22.6 (20.4, 24.8) | *0.03* | 27.2 (24.4, 30.0) | 28.8 (26.1, 31.5) | 26.4 (23.8, 28.9) | 25.3 (22.9, 27.6) | *0.36* |
| **Health insurance subsidy** |  |  |  |  |  |  |  |  |  |  |
| Full | 34.9 (33.1, 36.8) | 29.4 (27.7, 31.2) | 26.6 (25.0, 28.1) | 24.8 (23.3, 26.3) | *<0.01* | 30.4 (28.5, 32.2) | 27.9 (26.2, 29.6) | 25.7 (24.1, 27.3) | 25.1 (23.6, 26.7) | *0.01* |
| Partial | 38.3 (32.1, 44.5) | 27.2 (22.2, 32.1) | 28.1 (23.1, 33.1) | 26.4 (21.6, 31.2) | *0.05* | 33.9 (27.9, 40.0) | 32.3 (27.1, 37.5) | 24.0 (19.3, 28.7) | 25.3 (20.6, 30.1) | *0.03* |
| None | 50.7 (41.6, 59.9) | 32.6 (25.7, 39.5) | 30.2 (22.5, 37.9) | 33.5 (27.1, 40.0) | *0.04* | 40.8 (31.1, 50.4) | 30.1 (23.0, 37.2) | 31.8 (23.4, 40.1) | 30.6 (23.7, 37.5) | *0.45* |

Values are prevalence with 95% confidence intervals from Poisson regressions with robust standard errors, adjusted for all age, sex, and survey. CHF: Swiss Francs (1 CHF = 1.10 USD on 11 November 2023).

^a^ P-value for linear trend from Poisson regressions with robust standard errors, adjusted for sex, age, and survey year plus the interaction term between each predictor as binary/ordinal and survey period as continuous.

**Table S2 continued.** Trends in prevalence of cardiovascular risk factors by demographic and socioeconomic factors, Bus Santé 2008–2019

|  | **Overweight** | | | |  | **Obesity** |  |  |  |  |
| --- | --- | --- | --- | --- | --- | --- | --- | --- | --- | --- |
|  | 2008–2010 | 2011–2013 | 2014–2016 | 2017-2019 | *p-trend^a^* | 2008–2010 | 2011–2013 | 2014–2016 | 2017-2019 | *p-trend^a^* |
| **Overall** | 36.0 (34.1, 37.9) | 33.6 (31.9, 35.3) | 32.2 (30.6, 33.9) | 31.3 (29.8, 32.8) | *0.01* | 13.7 (12.3, 15.0) | 12.4 (11.2, 13.6) | 11.8 (10.6, 12.9) | 11.4 (10.3, 12.5) | *0.07* |
| **Age group**, y |  |  |  |  |  |  |  |  |  |  |
| ≥50 | 31.8 (29.2, 34.4) | 28.7 (26.6, 30.9) | 27.2 (25.2, 29.3) | 27.1 (25.2, 29.1) | *0.14* | 9.6 (7.9, 11.3) | 9.3 (7.9, 10.8) | 8.6 (7.3, 9.9) | 8.7 (7.4, 9.9) | *0.15* |
| <50 | 39.7 (37.1, 42.3) | 39.8 (37.2, 42.5) | 39.4 (36.8, 42.0) | 36.9 (34.5, 39.3) | *0.01* | 17.2 (15.2, 19.3) | 16.3 (14.2, 18.3) | 16.3 (14.2, 18.3) | 15.1 (13.2, 16.9) | *0.29* |
| **Sex** |  |  |  |  |  |  |  |  |  |  |
| Men | 45.2 (42.4, 48.0) | 42.6 (40.0, 45.1) | 39.6 (37.1, 42.0) | 39.8 (37.4, 42.1) | *0.01* | 15.0 (13.0, 17.0) | 13.7 (11.9, 15.4) | 12.6 (10.9, 14.2) | 12.8 (11.3, 14.4) | *0.26* |
| Women | 27.1 (24.6, 29.5) | 24.8 (22.6, 27.0) | 25.1 (23.0, 27.3) | 23.3 (21.3, 25.3) | *0.14* | 12.4 (10.5, 14.2) | 11.2 (9.5, 12.8) | 11.0 (9.4, 12.5) | 10.0 (8.6, 11.4) | *0.15* |
| **Educational level** |  |  |  |  |  |  |  |  |  |  |
| Primary/lower secondary | 40.8 (37.8, 43.8) | 38.0 (35.1, 40.9) | 37.6 (34.6, 40.6) | 36.2 (33.3, 39.1) | *0.11* | 17.5 (15.2, 19.9) | 16.9 (14.6, 19.1) | 17.9 (15.5, 20.3) | 16.7 (14.5, 19.0) | *0.82* |
| Secondary | 35.9 (31.9, 39.9) | 33.5 (30.0, 36.9) | 34.2 (30.9, 37.6) | 32.8 (29.5, 36.0) | *0.56* | 11.9 (9.2, 14.6) | 13.0 (10.5, 15.5) | 11.2 (9.0, 13.4) | 11.2 (9.0, 13.4) | *0.54* |
| Tertiary | 30.4 (27.3, 33.4) | 29.4 (26.8, 32.1) | 26.8 (24.4, 29.1) | 27.3 (25.1, 29.4) | *0.21* | 9.9 (7.9, 11.9) | 8.0 (6.4, 9.6) | 7.1 (5.7, 8.6) | 7.8 (6.4, 9.1) | *0.33* |
| **Household income**, CHF/month |  |  |  |  |  |  |  |  |  |  |
| <5000 | 36.8 (32.5, 41.0) | 34.4 (30.6, 38.3) | 32.0 (28.2, 35.7) | 32.2 (28.7, 35.8) | *0.21* | 21.9 (18.3, 25.5) | 16.1 (13.2, 19.0) | 16.7 (13.7, 19.6) | 14.5 (11.8, 17.1) | *0.02* |
| 5000-6999 | 35.8 (31.3, 40.2) | 36.8 (32.6, 41.0) | 35.2 (31.1, 39.3) | 35.7 (31.6, 39.8) | *0.98* | 13.8 (10.5, 17.1) | 15.6 (12.4, 18.7) | 15.3 (12.2, 18.4) | 12.5 (9.7, 15.3) | *0.59* |
| 7000-9499 | 40.0 (35.8, 44.2) | 36.6 (32.5, 40.7) | 35.3 (31.3, 39.3) | 33.8 (30.2, 37.5) | *0.07* | 12.5 (9.7, 15.4) | 10.4 (7.8, 13.1) | 10.8 (8.2, 13.4) | 12.7 (10.1, 15.3) | *0.62* |
| ≥9500 | 33.8 (30.8, 36.8) | 30.7 (28.0, 33.4) | 31.8 (29.1, 34.4) | 29.2 (26.8, 31.6) | *0.13* | 9.1 (7.2, 11.0) | 9.7 (7.8, 11.5) | 9.1 (7.4, 10.8) | 8.7 (7.1, 10.2) | *0.87* |
| **Health insurance subsidy** |  |  |  |  |  |  |  |  |  |  |
| Full | 35.8 (33.8, 37.8) | 33.8 (31.9, 35.7) | 32.6 (30.8, 34.4) | 31.3 (29.6, 32.9) | *0.01* | 12.6 (11.2, 14.0) | 11.6 (10.3, 12.9) | 11.2 (10.0, 12.4) | 10.7 (9.6, 11.8) | *0.16* |
| Partial | 39.4 (33.0, 45.8) | 35.8 (30.5, 41.1) | 33.6 (28.4, 38.7) | 33.2 (28.1, 38.4) | *0.20* | 16.7 (11.7, 21.6) | 15.1 (11.2, 19.1) | 14.2 (10.3, 18.1) | 11.7 (8.2, 15.2) | *0.17* |
| None | 33.4 (23.6, 43.2) | 30.7 (22.7, 38.7) | 28.8 (20.2, 37.4) | 32.2 (24.3, 40.1) | *0.94* | 29.3 (19.5, 39.0) | 21.4 (14.2, 28.5) | 23.9 (15.8, 32.0) | 21.1 (14.4, 27.7) | *0.47* |

Values are prevalence with 95% confidence intervals from Poisson regressions with robust standard errors, adjusted for all age, sex, and survey. CHF: Swiss Francs (1 CHF = 1.10 USD on 11 November 2023).

^a^ P-value for linear trend from Poisson regressions with robust standard errors, adjusted for sex, age, and survey year plus the interaction term between each predictor as binary/ordinal and survey period as continuous.

**Table S2 continued.** Trends in prevalence of cardiovascular risk factors by demographic and socioeconomic factors, Bus Santé 2008–2019

|  | **Hazardous alcohol use** | |  |  |  | **Diabetes** |  |  |  |  |
| --- | --- | --- | --- | --- | --- | --- | --- | --- | --- | --- |
|  | 2008–2010 | 2011–2013 | 2014–2016 | 2017-2019 | *p-trend^a^* | 2008–2010 | 2011–2013 | 2014–2016 | 2017-2019 | *p-trend^a^* |
| **Overall** | 26.0 (24.3, 27.7) | 22.2 (20.7, 23.7) | 20.0 (18.6, 21.4) | 19.6 (18.0, 21.2) | *<0.01* | 8.3 (7.2, 9.3) | 6.2 (5.3, 7.0) | 6.5 (5.7, 7.3) | 6.1 (5.4, 6.9) | *0.16* |
| **Age group**, y |  |  |  |  |  |  |  |  |  |  |
| ≥50 | 18.9 (16.7, 21.2) | 16.8 (15.0, 18.7) | 16.6 (14.9, 18.3) | 14.7 (12.8, 16.6) | *<0.01* | 4.2 (3.1, 5.4) | 2.6 (1.8, 3.4) | 2.9 (2.1, 3.6) | 2.5 (1.8, 3.2) | *0.74* |
| <50 | 32.3 (29.8, 34.8) | 29.1 (26.6, 31.6) | 25.0 (22.6, 27.4) | 26.3 (23.5, 29.0) | *0.01* | 11.9 (10.1, 13.6) | 10.8 (9.1, 12.5) | 11.7 (10.0, 13.5) | 11.1 (9.5, 12.7) | *0.02* |
| **Sex** |  |  |  |  |  |  |  |  |  |  |
| Men | 28.0 (25.5, 30.5) | 23.3 (21.1, 25.4) | 20.0 (18.0, 22.0) | 19.8 (17.5, 22.1) | *<0.01* | 10.0 (8.4, 11.7) | 7.3 (6.0, 8.6) | 8.0 (6.7, 9.3) | 6.8 (5.7, 8.0) | *0.14* |
| Women | 24.1 (21.8, 26.4) | 21.2 (19.1, 23.2) | 20.0 (18.1, 22.0) | 19.5 (17.3, 21.7) | *0.02* | 6.6 (5.3, 7.9) | 5.1 (4.0, 6.2) | 5.0 (4.0, 6.1) | 5.5 (4.5, 6.5) | *0.61* |
| **Educational level** |  |  |  |  |  |  |  |  |  |  |
| Primary/lower secondary | 28.2 (25.4, 30.9) | 21.7 (19.2, 24.2) | 21.5 (19.0, 24.0) | 19.3 (16.4, 22.1) | *<0.01* | 10.4 (8.6, 12.2) | 8.2 (6.6, 9.9) | 7.9 (6.4, 9.5) | 8.4 (6.9, 10.0) | *0.63* |
| Secondary | 24.3 (20.9, 27.7) | 22.6 (19.6, 25.6) | 19.2 (16.5, 21.9) | 18.0 (14.8, 21.2) | *0.02* | 8.2 (6.0, 10.4) | 5.6 (3.9, 7.3) | 6.1 (4.4, 7.7) | 5.7 (4.1, 7.2) | *0.33* |
| Tertiary | 25.1 (22.3, 28.0) | 22.5 (20.0, 25.0) | 19.6 (17.4, 21.7) | 20.2 (17.8, 22.7) | *0.01* | 5.8 (4.3, 7.3) | 4.3 (3.1, 5.6) | 5.5 (4.2, 6.7) | 4.7 (3.6, 5.7) | *0.91* |
| **Household income**, CHF/month |  |  |  |  |  |  |  |  |  |  |
| <5000 | 23.4 (19.8, 27.1) | 19.8 (16.6, 22.9) | 19.6 (16.4, 22.8) | 18.8 (15.3, 22.4) | *0.15* | 13.0 (10.2, 15.8) | 9.2 (7.0, 11.4) | 10.1 (7.8, 12.4) | 9.9 (7.8, 12.0) | *0.58* |
| 5000-6999 | 25.4 (21.3, 29.5) | 21.8 (18.3, 25.3) | 23.0 (19.4, 26.6) | 21.5 (17.3, 25.8) | *0.60* | 10.5 (7.7, 13.3) | 7.1 (4.9, 9.3) | 7.5 (5.3, 9.6) | 4.7 (2.9, 6.5) | *0.01* |
| 7000-9499 | 25.9 (22.3, 29.6) | 22.6 (19.1, 26.2) | 19.3 (16.0, 22.5) | 17.3 (13.8, 20.9) | *0.01* | 6.4 (4.3, 8.4) | 4.1 (2.4, 5.8) | 7.0 (5.0, 9.1) | 6.5 (4.7, 8.4) | *0.25* |
| ≥9500 | 26.8 (23.9, 29.7) | 23.9 (21.3, 26.6) | 20.0 (17.6, 22.4) | 21.1 (18.3, 24.0) | *0.01* | 5.1 (3.7, 6.5) | 4.6 (3.3, 5.9) | 4.6 (3.4, 5.8) | 4.8 (3.6, 6.0) | *0.97* |
| **Health insurance subsidy** |  |  |  |  |  |  |  |  |  |  |
| Full | 26.6 (24.7, 28.4) | 22.6 (20.9, 24.2) | 20.4 (18.8, 21.9) | 20.6 (18.8, 22.4) | *<0.01* | 7.3 (6.3, 8.4) | 5.7 (4.8, 6.6) | 6.3 (5.4, 7.2) | 5.6 (4.8, 6.5) | *0.37* |
| Partial | 21.3 (15.9, 26.6) | 19.1 (14.8, 23.5) | 21.9 (17.3, 26.4) | 15.3 (10.6, 20.0) | *0.38* | 14.5 (9.7, 19.2) | 9.0 (5.8, 12.3) | 5.8 (3.1, 8.4) | 8.1 (5.1, 11.1) | *0.04* |
| None | 22.4 (14.2, 30.7) | 20.6 (13.8, 27.5) | 12.2 (5.8, 18.7) | 14.3 (6.8, 21.8) | *0.06* | 16.5 (9.4, 23.5) | 10.4 (5.6, 15.3) | 16.4 (9.6, 23.1) | 12.9 (7.8, 18.1) | *0.98* |

Values are prevalence with 95% confidence intervals from Poisson regressions with robust standard errors, adjusted for all age, sex, and survey. CHF: Swiss Francs (1 CHF = 1.10 USD on 11 November 2023).

^a^ P-value for linear trend from Poisson regressions with robust standard errors, adjusted for sex, age, and survey year plus the interaction term between each predictor as binary/ordinal and survey period as continuous.

**Table S2 continued.** Trends in prevalence of cardiovascular risk factors by demographic and socioeconomic factors, Bus Santé 2008–2019

|  | **Smoking** | | | |  | **Sedentarity** |  |  |  |  |
| --- | --- | --- | --- | --- | --- | --- | --- | --- | --- | --- |
|  | 2008–2010 | 2011–2013 | 2014–2016 | 2017-2019 | *p-trend^a^* | 2008–2010 | 2011–2013 | 2014–2016 | 2017-2019 | *p-trend^a^* |
| **Overall** | 21.2 (19.6, 22.8) | 22.6 (21.1, 24.1) | 22.3 (20.9, 23.7) | 20.1 (18.8, 21.4) | *0.04* | 15.9 (14.5, 17.4) | 14.9 (13.6, 16.2) | 16.1 (14.8, 17.4) | 11.9 (10.6, 13.2) | *0.01* |
| **Age group**, y |  |  |  |  |  |  |  |  |  |  |
| ≥50 | 26.1 (23.6, 28.5) | 25.6 (23.5, 27.7) | 26.1 (24.2, 28.1) | 22.5 (20.7, 24.3) | *0.63* | 11.3 (9.5, 13.1) | 11.5 (10.0, 13.1) | 13.7 (12.1, 15.2) | 10.9 (9.2, 12.5) | *<0.01* |
| <50 | 16.8 (14.8, 18.8) | 18.7 (16.6, 20.9) | 16.7 (14.7, 18.7) | 16.7 (14.8, 18.6) | *0.02* | 20.1 (18.0, 22.3) | 19.3 (17.1, 21.4) | 19.7 (17.5, 21.9) | 13.3 (11.2, 15.4) | *0.71* |
| **Sex** |  |  |  |  |  |  |  |  |  |  |
| Men | 21.6 (19.3, 23.8) | 24.1 (21.9, 26.3) | 24.0 (21.9, 26.1) | 22.9 (20.9, 24.9) | *0.99* | 17.3 (15.2, 19.4) | 16.4 (14.5, 18.4) | 17.2 (15.3, 19.1) | 12.5 (10.6, 14.4) | *0.03* |
| Women | 20.8 (18.7, 23.0) | 21.2 (19.1, 23.3) | 20.7 (18.8, 22.6) | 17.5 (15.8, 19.2) | *0.01* | 14.6 (12.7, 16.6) | 13.4 (11.7, 15.2) | 15.1 (13.3, 16.8) | 11.3 (9.6, 13.1) | *0.18* |
| **Educational level** |  |  |  |  |  |  |  |  |  |  |
| Primary/lower secondary | 26.5 (23.8, 29.2) | 28.4 (25.6, 31.2) | 30.0 (27.2, 32.9) | 26.9 (24.2, 29.7) | *0.97* | 22.2 (19.7, 24.8) | 19.2 (16.8, 21.6) | 21.4 (18.9, 23.9) | 15.4 (12.7, 18.1) | *0.01* |
| Secondary | 22.6 (19.4, 25.9) | 25.6 (22.5, 28.7) | 23.0 (20.1, 25.9) | 21.5 (18.7, 24.2) | *0.11* | 13.4 (10.7, 16.1) | 13.5 (11.0, 16.0) | 15.1 (12.7, 17.6) | 14.1 (11.2, 17.0) | *0.28* |
| Tertiary | 14.7 (12.4, 17.0) | 15.5 (13.4, 17.6) | 16.2 (14.3, 18.1) | 15.4 (13.7, 17.1) | *0.98* | 10.6 (8.6, 12.7) | 11.5 (9.6, 13.4) | 12.6 (10.8, 14.4) | 8.3 (6.7, 10.0) | *0.41* |
| **Household income**, CHF/month |  |  |  |  |  |  |  |  |  |  |
| <5000 | 30.4 (26.4, 34.5) | 32.0 (28.3, 35.6) | 30.2 (26.6, 33.8) | 28.6 (25.2, 31.9) | *0.22* | 19.4 (16.0, 22.8) | 14.7 (11.9, 17.5) | 21.5 (18.2, 24.8) | 16.4 (13.1, 19.8) | *0.90* |
| 5000-6999 | 22.3 (18.5, 26.2) | 21.6 (18.0, 25.2) | 27.3 (23.6, 31.0) | 21.3 (17.8, 24.7) | *0.93* | 19.0 (15.4, 22.7) | 17.8 (14.4, 21.1) | 18.6 (15.2, 21.9) | 14.5 (10.8, 18.2) | *0.22* |
| 7000-9499 | 21.0 (17.6, 24.3) | 20.8 (17.4, 24.3) | 22.3 (18.8, 25.7) | 19.7 (16.6, 22.9) | *0.54* | 15.6 (12.5, 18.6) | 15.3 (12.2, 18.4) | 14.9 (12.0, 17.9) | 12.1 (9.0, 15.2) | *0.22* |
| ≥9500 | 16.7 (14.3, 19.1) | 17.1 (14.9, 19.4) | 16.2 (14.1, 18.3) | 14.6 (12.8, 16.5) | *0.06* | 12.3 (10.1, 14.4) | 12.3 (10.3, 14.3) | 12.2 (10.3, 14.2) | 7.6 (5.8, 9.3) | *0.01* |
| **Health insurance subsidy** |  |  |  |  |  |  |  |  |  |  |
| Full | 20.1 (18.4, 21.7) | 20.3 (18.7, 21.9) | 20.9 (19.3, 22.4) | 18.8 (17.3, 20.2) | *0.08* | 15.9 (14.4, 17.5) | 15.2 (13.8, 16.6) | 16.3 (14.8, 17.7) | 11.5 (10.1, 12.9) | *0.01* |
| Partial | 25.0 (19.8, 30.1) | 28.2 (23.6, 32.8) | 26.1 (21.8, 30.4) | 24.4 (19.9, 28.8) | *0.41* | 15.4 (10.7, 20.1) | 14.2 (10.4, 18.0) | 16.3 (12.4, 20.3) | 12.6 (8.4, 16.8) | *0.91* |
| None | 35.8 (26.0, 45.5) | 42.0 (33.1, 50.9) | 39.1 (29.1, 49.1) | 33.0 (24.8, 41.1) | *0.33* | 19.3 (11.5, 27.2) | 13.6 (7.9, 19.4) | 18.3 (10.7, 25.9) | 10.4 (4.0, 16.8) | *0.18* |

Values are prevalence with 95% confidence intervals from Poisson regressions with robust standard errors, adjusted for all age, sex, and survey. CHF: Swiss Francs (1 CHF = 1.10 USD on 11 November 2023).

^a^ P-value for linear trend from Poisson regressions with robust standard errors, adjusted for sex, age, and survey year plus the interaction term between each predictor as binary/ordinal and survey period as continuous.

**Table S3.** Trends in the association of cardiovascular risk factors prevalence with demographic and socioeconomic factors, Bus Santé 2008–2019

|  | **Hypertension** | | | |  | **Hypercholesterolemia** | |  |  |  |
| --- | --- | --- | --- | --- | --- | --- | --- | --- | --- | --- |
|  | 2008–2010 | 2011–2013 | 2014–2016 | 2017-2019 | *p-trend^a^* | 2008–2010 | 2011–2013 | 2014–2016 | 2017-2019 | *p-trend^a^* |
| **Age group**, y |  |  |  |  |  |  |  |  |  |  |
| ≥50 | 2.34 (2.06, 2.66) | 2.78 (2.43, 3.18) | 2.77 (2.42, 3.16) | 2.65 (2.32, 3.02) | *0.33* | 1.96 (1.72, 2.24) | 2.45 (2.15, 2.80) | 2.37 (2.07, 2.70) | 2.23 (1.97, 2.54) | *0.23* |
| <50 | 1.00 (ref.) | 1.00 (ref.) | 1.00 (ref.) | 1.00 (ref.) |  | 1.00 (ref.) | 1.00 (ref.) | 1.00 (ref.) | 1.00 (ref.) |  |
| **Sex** |  |  |  |  |  |  |  |  |  |  |
| Men | 1.41 (1.27, 1.56) | 1.34 (1.19, 1.50) | 1.52 (1.35, 1.71) | 1.38 (1.23, 1.55) | *0.56* | 1.55 (1.37, 1.75) | 1.35 (1.20, 1.53) | 1.25 (1.11, 1.41) | 1.28 (1.14, 1.44) | *0.03* |
| Women | 1.00 (ref.) | 1.00 (ref.) | 1.00 (ref.) | 1.00 (ref.) |  | 1.00 (ref.) | 1.00 (ref.) | 1.00 (ref.) | 1.00 (ref.) |  |
| **Educational level** |  |  |  |  |  |  |  |  |  |  |
| Primary/lower secondary | 1.19 (1.04, 1.35) | 1.04 (0.91, 1.20) | 1.30 (1.13, 1.50) | 1.22 (1.07, 1.40) | *0.76* | 0.98 (0.85, 1.13) | 1.12 (0.97, 1.29) | 1.18 (1.02, 1.37) | 1.30 (1.13, 1.49) | *0.04* |
| Secondary | 1.07 (0.92, 1.25) | 1.07 (0.92, 1.24) | 1.25 (1.08, 1.46) | 1.06 (0.91, 1.24) | *0.97* | 0.86 (0.73, 1.02) | 1.05 (0.90, 1.23) | 1.07 (0.91, 1.25) | 1.09 (0.93, 1.27) | *0.09* |
| Tertiary | 1.00 (ref.) | 1.00 (ref.) | 1.00 (ref.) | 1.00 (ref.) |  | 1.00 (ref.) | 1.00 (ref.) | 1.00 (ref.) | 1.00 (ref.) |  |
| **Household income**, CHF/month |  |  |  |  |  |  |  |  |  |  |
| <5000 | 1.46 (1.25, 1.71) | 1.24 (1.04, 1.47) | 0.94 (0.79, 1.12) | 1.18 (0.99, 1.39) | *0.03* | 1.33 (1.11, 1.58) | 0.95 (0.79, 1.13) | 0.93 (0.78, 1.11) | 0.88 (0.74, 1.05) | *0.03* |
| 5000-6999 | 1.11 (0.94, 1.32) | 1.26 (1.08, 1.48) | 1.06 (0.90, 1.24) | 1.11 (0.93, 1.32) | *0.97* | 1.08 (0.90, 1.29) | 0.96 (0.81, 1.14) | 1.02 (0.86, 1.20) | 1.04 (0.88, 1.23) | *0.38* |
| 7000-9499 | 1.31 (1.13, 1.52) | 1.06 (0.90, 1.26) | 1.03 (0.87, 1.21) | 1.12 (0.96, 1.32) | *0.23* | 1.20 (1.02, 1.41) | 0.91 (0.77, 1.07) | 0.94 (0.79, 1.12) | 0.98 (0.84, 1.16) | *0.48* |
| ≥9500 | 1.00 (ref.) | 1.00 (ref.) | 1.00 (ref.) | 1.00 (ref.) |  | 1.00 (ref.) | 1.00 (ref.) | 1.00 (ref.) | 1.00 (ref.) |  |
| **Health insurance subsidy** |  |  |  |  |  |  |  |  |  |  |
| Full | 1.22 (0.99, 1.50) | 0.95 (0.74, 1.22) | 1.17 (0.87, 1.57) | 1.24 (0.99, 1.55) | *0.95* | 1.15 (0.87, 1.50) | 1.07 (0.81, 1.41) | 1.24 (0.91, 1.69) | 1.27 (0.98, 1.64) | *0.12* |
| Partial | 0.98 (0.82, 1.18) | 0.85 (0.69, 1.06) | 0.98 (0.79, 1.22) | 1.04 (0.85, 1.27) | *0.67* | 1.00 (0.81, 1.24) | 1.12 (0.92, 1.37) | 0.96 (0.78, 1.19) | 0.91 (0.73, 1.14) | *0.99* |
| None | 1.00 (ref.) | 1.00 (ref.) | 1.00 (ref.) | 1.00 (ref.) |  | 1.00 (ref.) | 1.00 (ref.) | 1.00 (ref.) | 1.00 (ref.) |  |

Values are prevalence ratios with 95% confidence intervals from Poisson regressions with robust standard errors, adjusted for all age, sex, and survey. CHF: Swiss Francs (1 CHF = 1.10 USD on 11 November 2023).

^a^ P-value for linear trend from Poisson regressions with robust standard errors, adjusted for sex, age, and survey year plus the interaction term between each predictor as binary/ordinal and survey period as continuous.

**Table S3 continued.** Trends in the association of cardiovascular risk factors prevalence with demographic and socioeconomic factors, Bus Santé 2008–2019

|  | **Overweight** | | | |  | **Obesity** |  |  |  |  |
| --- | --- | --- | --- | --- | --- | --- | --- | --- | --- | --- |
|  | 2008–2010 | 2011–2013 | 2014–2016 | 2017-2019 | *p-trend^a^* | 2008–2010 | 2011–2013 | 2014–2016 | 2017-2019 | *p-trend^a^* |
| **Age group**, y |  |  |  |  |  |  |  |  |  |  |
| ≥50 | 1.26 (1.13, 1.41) | 1.37 (1.23, 1.52) | 1.36 (1.22, 1.52) | 1.28 (1.16, 1.42) | *0.85* | 1.60 (1.27, 2.00) | 1.53 (1.23, 1.89) | 1.67 (1.35, 2.05) | 1.50 (1.22, 1.85) | *0.75* |
| <50 | 1.00 (ref.) | 1.00 (ref.) | 1.00 (ref.) | 1.00 (ref.) |  | 1.00 (ref.) | 1.00 (ref.) | 1.00 (ref.) | 1.00 (ref.) |  |
| **Sex** |  |  |  |  |  |  |  |  |  |  |
| Men | 1.68 (1.50, 1.89) | 1.77 (1.57, 1.98) | 1.61 (1.44, 1.80) | 1.71 (1.54, 1.91) | *0.85* | 1.30 (1.05, 1.60) | 1.25 (1.02, 1.54) | 1.24 (1.02, 1.52) | 1.32 (1.08, 1.61) | *0.73* |
| Women | 1.00 (ref.) | 1.00 (ref.) | 1.00 (ref.) | 1.00 (ref.) |  | 1.00 (ref.) | 1.00 (ref.) | 1.00 (ref.) | 1.00 (ref.) |  |
| **Educational level** |  |  |  |  |  |  |  |  |  |  |
| Primary/lower secondary | 1.39 (1.22, 1.60) | 1.23 (1.08, 1.41) | 1.42 (1.24, 1.62) | 1.33 (1.17, 1.51) | *0.91* | 1.39 (1.08, 1.80) | 2.02 (1.53, 2.67) | 2.02 (1.55, 2.63) | 1.88 (1.46, 2.42) | *0.38* |
| Secondary | 1.23 (1.05, 1.44) | 1.10 (0.95, 1.27) | 1.34 (1.16, 1.54) | 1.24 (1.09, 1.42) | *0.39* | 1.02 (0.74, 1.40) | 1.67 (1.23, 2.25) | 1.47 (1.10, 1.97) | 1.27 (0.94, 1.71) | *0.75* |
| Tertiary | 1.00 (ref.) | 1.00 (ref.) | 1.00 (ref.) | 1.00 (ref.) |  | 1.00 (ref.) | 1.00 (ref.) | 1.00 (ref.) | 1.00 (ref.) |  |
| **Household income**, CHF/month |  |  |  |  |  |  |  |  |  |  |
| <5000 | 0.95 (0.81, 1.13) | 1.04 (0.88, 1.23) | 0.89 (0.76, 1.06) | 0.99 (0.84, 1.16) | *0.98* | 2.10 (1.55, 2.84) | 1.21 (0.89, 1.65) | 1.34 (0.99, 1.81) | 1.21 (0.90, 1.64) | *0.06* |
| 5000-6999 | 0.93 (0.79, 1.10) | 1.10 (0.95, 1.29) | 0.97 (0.83, 1.13) | 1.10 (0.95, 1.28) | *0.26* | 1.46 (1.05, 2.02) | 1.28 (0.96, 1.72) | 1.32 (0.99, 1.77) | 1.24 (0.91, 1.69) | *0.84* |
| 7000-9499 | 1.10 (0.95, 1.27) | 1.12 (0.96, 1.30) | 1.00 (0.86, 1.15) | 1.10 (0.95, 1.26) | *0.73* | 1.35 (0.98, 1.87) | 0.91 (0.66, 1.26) | 1.05 (0.77, 1.42) | 1.28 (0.95, 1.70) | *0.72* |
| ≥9500 | 1.00 (ref.) | 1.00 (ref.) | 1.00 (ref.) | 1.00 (ref.) |  | 1.00 (ref.) | 1.00 (ref.) | 1.00 (ref.) | 1.00 (ref.) |  |
| **Health insurance subsidy** |  |  |  |  |  |  |  |  |  |  |
| Full | 0.94 (0.68, 1.30) | 0.86 (0.63, 1.16) | 0.86 (0.61, 1.21) | 1.03 (0.78, 1.35) | *0.66* | 1.56 (1.06, 2.29) | 1.35 (0.90, 2.04) | 1.56 (1.03, 2.36) | 1.61 (1.09, 2.37) | *0.52* |
| Partial | 1.09 (0.91, 1.31) | 1.03 (0.86, 1.23) | 1.03 (0.86, 1.24) | 1.02 (0.85, 1.22) | *0.59* | 1.00 (0.72, 1.40) | 1.05 (0.77, 1.45) | 1.08 (0.79, 1.49) | 0.99 (0.70, 1.40) | *0.49* |
| None | 1.00 (ref.) | 1.00 (ref.) | 1.00 (ref.) | 1.00 (ref.) |  | 1.00 (ref.) | 1.00 (ref.) | 1.00 (ref.) | 1.00 (ref.) |  |

Values are prevalence ratios with 95% confidence intervals from Poisson regressions with robust standard errors, adjusted for all age, sex, and survey. CHF: Swiss Francs (1 CHF = 1.10 USD on 11 November 2023).

^a^ P-value for linear trend from Poisson regressions with robust standard errors, adjusted for sex, age, and survey year plus the interaction term between each predictor as binary/ordinal and survey period as continuous.

**Table S3 continued.** Trends in the association of cardiovascular risk factors prevalence with demographic and socioeconomic factors, Bus Santé 2008–2019

|  | **Hazardous alcohol use** | | | |  | **Diabetes** |  |  |  |  |
| --- | --- | --- | --- | --- | --- | --- | --- | --- | --- | --- |
|  | 2008–2010 | 2011–2013 | 2014–2016 | 2017-2019 | *p-trend^a^* | 2008–2010 | 2011–2013 | 2014–2016 | 2017-2019 | *p-trend^a^* |
| **Age group**, y |  |  |  |  |  |  |  |  |  |  |
| ≥50 | 1.67 (1.44, 1.94) | 1.82 (1.56, 2.12) | 1.43 (1.23, 1.67) | 1.71 (1.43, 2.05) | *0.40* | 2.63 (1.91, 3.63) | 3.47 (2.42, 4.98) | 3.61 (2.62, 4.99) | 3.89 (2.81, 5.40) | *0.09* |
| <50 | 1.00 (ref.) | 1.00 (ref.) | 1.00 (ref.) | 1.00 (ref.) |  | 1.00 (ref.) | 1.00 (ref.) | 1.00 (ref.) | 1.00 (ref.) |  |
| **Sex** |  |  |  |  |  |  |  |  |  |  |
| Men | 1.18 (1.02, 1.35) | 1.11 (0.96, 1.29) | 0.99 (0.85, 1.15) | 1.01 (0.85, 1.20) | *0.06* | 1.77 (1.35, 2.32) | 1.41 (1.03, 1.92) | 1.90 (1.42, 2.54) | 1.17 (0.90, 1.54) | *0.19* |
| Women | 1.00 (ref.) | 1.00 (ref.) | 1.00 (ref.) | 1.00 (ref.) |  | 1.00 (ref.) | 1.00 (ref.) | 1.00 (ref.) | 1.00 (ref.) |  |
| **Educational level** |  |  |  |  |  |  |  |  |  |  |
| Primary/lower secondary | 1.23 (1.05, 1.46) | 1.04 (0.87, 1.25) | 1.16 (0.96, 1.39) | 0.99 (0.80, 1.22) | *0.24* | 1.31 (0.94, 1.82) | 1.62 (1.13, 2.34) | 1.24 (0.88, 1.76) | 1.57 (1.15, 2.14) | *0.91* |
| Secondary | 1.01 (0.83, 1.22) | 1.02 (0.84, 1.23) | 1.04 (0.85, 1.26) | 0.82 (0.64, 1.04) | *0.43* | 1.13 (0.76, 1.67) | 1.25 (0.82, 1.89) | 1.09 (0.74, 1.60) | 1.12 (0.77, 1.63) | *0.71* |
| Tertiary | 1.00 (ref.) | 1.00 (ref.) | 1.00 (ref.) | 1.00 (ref.) |  | 1.00 (ref.) | 1.00 (ref.) | 1.00 (ref.) | 1.00 (ref.) |  |
| **Household income**, CHF/month |  |  |  |  |  |  |  |  |  |  |
| <5000 | 0.84 (0.68, 1.05) | 0.82 (0.65, 1.04) | 0.98 (0.78, 1.24) | 0.99 (0.76, 1.28) | *0.43* | 1.95 (1.31, 2.89) | 1.49 (0.97, 2.30) | 1.90 (1.25, 2.90) | 1.57 (1.08, 2.26) | *0.65* |
| 5000-6999 | 0.92 (0.75, 1.12) | 0.93 (0.76, 1.15) | 1.08 (0.88, 1.34) | 1.08 (0.84, 1.38) | *0.29* | 1.87 (1.26, 2.79) | 1.31 (0.86, 2.01) | 1.55 (1.02, 2.36) | 0.88 (0.55, 1.39) | *0.04* |
| 7000-9499 | 0.92 (0.77, 1.11) | 0.95 (0.77, 1.16) | 0.93 (0.75, 1.15) | 0.81 (0.63, 1.05) | *0.35* | 1.16 (0.75, 1.79) | 0.73 (0.44, 1.23) | 1.45 (0.96, 2.19) | 1.25 (0.85, 1.82) | *0.32* |
| ≥9500 | 1.00 (ref.) | 1.00 (ref.) | 1.00 (ref.) | 1.00 (ref.) |  | 1.00 (ref.) | 1.00 (ref.) | 1.00 (ref.) | 1.00 (ref.) |  |
| **Health insurance subsidy** |  |  |  |  |  |  |  |  |  |  |
| Full | 0.96 (0.65, 1.42) | 1.04 (0.72, 1.52) | 0.64 (0.36, 1.13) | 0.75 (0.44, 1.29) | *0.39* | 1.73 (1.07, 2.78) | 1.17 (0.66, 2.08) | 2.02 (1.24, 3.30) | 1.55 (0.94, 2.55) | *0.05* |
| Partial | 0.81 (0.60, 1.08) | 0.86 (0.65, 1.13) | 1.05 (0.82, 1.35) | 0.75 (0.54, 1.06) | *0.36* | 1.51 (1.03, 2.23) | 1.19 (0.76, 1.86) | 0.80 (0.49, 1.31) | 1.02 (0.66, 1.57) | *0.95* |
| None | 1.00 (ref.) | 1.00 (ref.) | 1.00 (ref.) | 1.00 (ref.) |  | 1.00 (ref.) | 1.00 (ref.) | 1.00 (ref.) | 1.00 (ref.) |  |

Values are prevalence ratios with 95% confidence intervals from Poisson regressions with robust standard errors, adjusted for all age, sex, and survey. CHF: Swiss Francs (1 CHF = 1.10 USD on 11 November 2023).

^a^ P-value for linear trend from Poisson regressions with robust standard errors, adjusted for sex, age, and survey year plus the interaction term between each predictor as binary/ordinal and survey period as continuous.

**Table S3 continued.** Trends in the association of cardiovascular risk factors prevalence with demographic and socioeconomic factors, Bus Santé 2008–2019

|  | **Smoking** | | | |  | **Sedentarity** |  |  |  |  |
| --- | --- | --- | --- | --- | --- | --- | --- | --- | --- | --- |
|  | 2008–2010 | 2011–2013 | 2014–2016 | 2017-2019 | *p-trend^a^* | 2008–2010 | 2011–2013 | 2014–2016 | 2017-2019 | *p-trend^a^* |
| **Age group**, y |  |  |  |  |  |  |  |  |  |  |
| ≥50 | 0.60 (0.51, 0.70) | 0.67 (0.57, 0.78) | 0.59 (0.50, 0.68) | 0.66 (0.57, 0.77) | *0.52* | 1.66 (1.35, 2.04) | 1.47 (1.21, 1.78) | 1.34 (1.13, 1.59) | 1.06 (0.82, 1.36) | *0.01* |
| <50 | 1.00 (ref.) | 1.00 (ref.) | 1.00 (ref.) | 1.00 (ref.) |  | 1.00 (ref.) | 1.00 (ref.) | 1.00 (ref.) | 1.00 (ref.) |  |
| **Sex** |  |  |  |  |  |  |  |  |  |  |
| Men | 1.05 (0.90, 1.22) | 1.17 (1.01, 1.36) | 1.19 (1.04, 1.37) | 1.26 (1.09, 1.45) | *0.09* | 1.20 (1.00, 1.45) | 1.21 (1.00, 1.47) | 1.15 (0.97, 1.37) | 1.13 (0.89, 1.44) | *0.38* |
| Women | 1.00 (ref.) | 1.00 (ref.) | 1.00 (ref.) | 1.00 (ref.) |  | 1.00 (ref.) | 1.00 (ref.) | 1.00 (ref.) | 1.00 (ref.) |  |
| **Educational level** |  |  |  |  |  |  |  |  |  |  |
| Primary/lower secondary | 1.59 (1.30, 1.95) | 1.58 (1.30, 1.92) | 1.71 (1.43, 2.04) | 1.64 (1.38, 1.96) | *0.42* | 2.01 (1.58, 2.56) | 1.52 (1.21, 1.92) | 1.44 (1.16, 1.79) | 1.79 (1.32, 2.42) | *0.57* |
| Secondary | 1.48 (1.19, 1.84) | 1.51 (1.23, 1.84) | 1.36 (1.12, 1.64) | 1.44 (1.19, 1.74) | *0.73* | 1.16 (0.86, 1.56) | 1.16 (0.89, 1.52) | 1.08 (0.85, 1.38) | 1.64 (1.18, 2.28) | *0.09* |
| Tertiary | 1.00 (ref.) | 1.00 (ref.) | 1.00 (ref.) | 1.00 (ref.) |  | 1.00 (ref.) | 1.00 (ref.) | 1.00 (ref.) | 1.00 (ref.) |  |
| **Household income**, CHF/month |  |  |  |  |  |  |  |  |  |  |
| <5000 | 1.50 (1.20, 1.87) | 1.41 (1.14, 1.74) | 1.43 (1.16, 1.76) | 1.57 (1.28, 1.93) | *0.82* | 1.27 (0.95, 1.69) | 1.13 (0.84, 1.53) | 1.66 (1.28, 2.14) | 2.08 (1.47, 2.94) | *0.07* |
| 5000-6999 | 1.15 (0.90, 1.45) | 1.07 (0.85, 1.34) | 1.36 (1.11, 1.67) | 1.27 (1.02, 1.58) | *0.24* | 1.24 (0.94, 1.62) | 1.35 (1.03, 1.77) | 1.34 (1.04, 1.74) | 1.81 (1.26, 2.61) | *0.27* |
| 7000-9499 | 1.16 (0.93, 1.44) | 1.08 (0.86, 1.35) | 1.22 (0.99, 1.51) | 1.22 (0.99, 1.51) | *0.51* | 1.08 (0.83, 1.41) | 1.17 (0.89, 1.53) | 1.14 (0.87, 1.48) | 1.43 (1.00, 2.06) | *0.41* |
| ≥9500 | 1.00 (ref.) | 1.00 (ref.) | 1.00 (ref.) | 1.00 (ref.) |  | 1.00 (ref.) | 1.00 (ref.) | 1.00 (ref.) | 1.00 (ref.) |  |
| **Health insurance subsidy** |  |  |  |  |  |  |  |  |  |  |
| Full | 1.33 (0.98, 1.81) | 1.66 (1.29, 2.12) | 1.36 (0.99, 1.86) | 1.27 (0.95, 1.70) | *0.75* | 0.98 (0.62, 1.53) | 0.74 (0.43, 1.26) | 0.78 (0.48, 1.26) | 0.49 (0.24, 1.01) | *0.79* |
| Partial | 1.06 (0.84, 1.34) | 1.14 (0.92, 1.40) | 1.10 (0.91, 1.34) | 1.01 (0.81, 1.27) | *0.48* | 0.87 (0.62, 1.22) | 0.84 (0.61, 1.16) | 0.77 (0.58, 1.03) | 0.75 (0.50, 1.13) | *0.28* |
| None | 1.00 (ref.) | 1.00 (ref.) | 1.00 (ref.) | 1.00 (ref.) |  | 1.00 (ref.) | 1.00 (ref.) | 1.00 (ref.) | 1.00 (ref.) |  |

Values are prevalence ratios with 95% confidence intervals from Poisson regressions with robust standard errors, adjusted for all age, sex, and survey. CHF: Swiss Francs (1 CHF = 1.10 USD on 11 November 2023).

^a^ P-value for linear trend from Poisson regressions with robust standard errors, adjusted for sex, age, and survey year plus the interaction term between each predictor as binary/ordinal and survey period as continuous.

**Table S4.** Trends in relative socioeconomic inequalities in cardiovascular risk factor prevalence, Bus Santé 2008–2019

|  | **2008–2010** | **2011–2013** | **2014–2016** | **2017-2019** | *p-trend^a^* |
| --- | --- | --- | --- | --- | --- |
| **Hypertension** |  |  |  |  |  |
| Educational level | 1.82 (1.62, 2.04) | 1.46 (1.29, 1.66) | 1.44 (1.26, 1.63) | 1.27 (1.12, 1.46) | *<0.01* |
| Household income | 1.92 (1.69, 2.18) | 1.49 (1.30, 1.71) | 1.35 (1.17, 1.56) | 1.27 (1.10, 1.47) | *<0.01* |
| Health insurance subsidy | 1.65 (1.39, 1.95) | 1.24 (1.05, 1.47) | 1.14 (0.95, 1.37) | 1.02 (0.85, 1.22) | *<0.01* |
| **Hypercholesterolemia** |  |  |  |  |  |
| Educational level | 1.32 (1.16, 1.50) | 1.29 (1.13, 1.47) | 1.16 (1.01, 1.33) | 1.15 (1.00, 1.32) | *0.01* |
| Household income | 1.31 (1.13, 1.52) | 1.20 (1.04, 1.39) | 1.09 (0.94, 1.27) | 1.01 (0.87, 1.18) | *<0.01* |
| Health insurance subsidy | 1.39 (1.16, 1.68) | 1.31 (1.10, 1.57) | 1.13 (0.93, 1.36) | 1.09 (0.90, 1.31) | *<0.01* |
| **Overweight** |  |  |  |  |  |
| Educational level | 1.72 (1.54, 1.93) | 1.62 (1.44, 1.82) | 1.62 (1.44, 1.83) | 1.53 (1.36, 1.73) | *0.06* |
| Household income | 1.27 (1.11, 1.45) | 1.20 (1.05, 1.37) | 1.13 (0.99, 1.30) | 1.10 (0.97, 1.26) | *0.04* |
| Health insurance subsidy | 1.18 (0.99, 1.40) | 1.08 (0.91, 1.27) | 1.01 (0.85, 1.21) | 0.97 (0.81, 1.15) | *0.01* |
| **Obesity** |  |  |  |  |  |
| Educational level | 3.41 (2.72, 4.28) | 3.49 (2.78, 4.38) | 3.58 (2.84, 4.51) | 3.32 (2.63, 4.19) | *0.88* |
| Household income | 3.16 (2.46, 4.05) | 2.68 (2.09, 3.43) | 2.78 (2.17, 3.56) | 2.37 (1.85, 3.04) | *0.03* |
| Health insurance subsidy | 2.65 (1.97, 3.56) | 2.34 (1.76, 3.11) | 2.30 (1.72, 3.09) | 1.98 (1.48, 2.66) | *0.03* |
| **Hazardous alcohol use** |  |  |  |  |  |
| Educational level | 1.34 (1.14, 1.56) | 1.07 (0.91, 1.27) | 0.97 (0.82, 1.16) | 0.87 (0.71, 1.06) | *<0.01* |
| Household income | 1.05 (0.87, 1.26) | 0.87 (0.72, 1.05) | 0.81 (0.67, 0.99) | 0.75 (0.60, 0.93) | *<0.01* |
| Health insurance subsidy | 0.89 (0.69, 1.14) | 0.73 (0.56, 0.93) | 0.64 (0.49, 0.84) | 0.57 (0.43, 0.76) | *<0.01* |
| **Diabetes** |  |  |  |  |  |
| Educational level | 2.78 (2.07, 3.74) | 2.23 (1.62, 3.06) | 2.40 (1.76, 3.27) | 2.39 (1.75, 3.27) | *0.41* |
| Household income | 4.24 (3.03, 5.95) | 2.93 (2.05, 4.21) | 3.64 (2.59, 5.12) | 3.18 (2.25, 4.48) | *0.20* |
| Health insurance subsidy | 3.98 (2.73, 5.80) | 2.66 (1.81, 3.91) | 3.05 (2.09, 4.45) | 2.77 (1.89, 4.05) | *0.07* |
| **Smoking** |  |  |  |  |  |
| Educational level | 2.54 (2.16, 2.97) | 2.73 (2.34, 3.19) | 2.71 (2.32, 3.16) | 2.42 (2.06, 2.84) | *0.54* |
| Household income | 2.77 (2.31, 3.33) | 2.73 (2.30, 3.25) | 2.84 (2.39, 3.37) | 2.37 (1.99, 2.84) | *0.13* |
| Health insurance subsidy | 2.27 (1.84, 2.79) | 2.34 (1.93, 2.84) | 2.24 (1.84, 2.72) | 1.91 (1.56, 2.35) | *0.06* |
| **Sedentarity** |  |  |  |  |  |
| Educational level | 2.81 (2.29, 3.46) | 2.55 (2.07, 3.14) | 3.02 (2.47, 3.70) | 1.95 (1.52, 2.51) | *0.01* |
| Household income | 2.39 (1.89, 3.03) | 1.99 (1.58, 2.52) | 2.65 (2.12, 3.32) | 1.69 (1.28, 2.23) | *0.07* |
| Health insurance subsidy | 1.08 (0.79, 1.47) | 0.99 (0.74, 1.33) | 1.23 (0.92, 1.64) | 0.64 (0.44, 0.91) | *<0.01* |

Values are RII (Relative Index of Inequality) with 95% confidence intervals from generalized linear models with robust standard errors, adjusted for age, sex, and survey year. CHF: Swiss Francs (1 CHF = 1.10 USD on 11 November 2023). RII = 1.00 indicates no difference in prevalence between most disadvantaged and most privileged socioeconomic groups.

^a^ P-value for linear trend from interaction term between each predictor as binary/ordinal and survey period as continuous.

**Table S5.** Trends in absolute socioeconomic inequalities in cardiovascular risk factor prevalence, Bus Santé 2008–2019

|  | **2008–2010** | **2011–2013** | **2014–2016** | **2017-2019** | *p-trend^a^* |
| --- | --- | --- | --- | --- | --- |
| **Hypertension** |  |  |  |  |  |
| Educational level | 0.19 (0.15, 0.23) | 0.11 (0.07, 0.14) | 0.10 (0.06, 0.14) | 0.06 (0.03, 0.10) | *<0.01* |
| Household income | 0.21 (0.17, 0.26) | 0.11 (0.07, 0.15) | 0.08 (0.04, 0.12) | 0.07 (0.02, 0.11) | *<0.01* |
| Health insurance subsidy | 0.16 (0.11, 0.22) | 0.06 (0.01, 0.11) | 0.04 (-0.01, 0.09) | 0.01 (-0.04, 0.06) | *<0.01* |
| **Hypercholesterolemia** |  |  |  |  |  |
| Educational level | 0.08 (0.04, 0.12) | 0.07 (0.03, 0.11) | 0.04 (0.00, 0.08) | 0.04 (0.00, 0.07) | *0.01* |
| Household income | 0.08 (0.03, 0.12) | 0.05 (0.01, 0.09) | 0.02 (-0.02, 0.06) | 0.00 (-0.04, 0.04) | *<0.01* |
| Health insurance subsidy | 0.10 (0.04, 0.15) | 0.08 (0.02, 0.13) | 0.03 (-0.02, 0.09) | 0.03 (-0.03, 0.08) | *<0.01* |
| **Overweight** |  |  |  |  |  |
| Educational level | 0.18 (0.14, 0.23) | 0.16 (0.12, 0.20) | 0.16 (0.12, 0.20) | 0.14 (0.10, 0.18) | *0.06* |
| Household income | 0.08 (0.03, 0.13) | 0.06 (0.01, 0.10) | 0.04 (0.00, 0.09) | 0.03 (-0.01, 0.08) | *0.04* |
| Health insurance subsidy | 0.06 (0.00, 0.12) | 0.02 (-0.03, 0.08) | 0.01 (-0.05, 0.06) | -0.01 (-0.07, 0.05) | *0.01* |
| **Obesity** |  |  |  |  |  |
| Educational level | 0.15 (0.12, 0.18) | 0.15 (0.12, 0.18) | 0.15 (0.12, 0.18) | 0.14 (0.11, 0.17) | *0.84* |
| Household income | 0.15 (0.11, 0.19) | 0.12 (0.09, 0.15) | 0.13 (0.09, 0.16) | 0.10 (0.07, 0.13) | *0.03* |
| Health insurance subsidy | 0.13 (0.09, 0.18) | 0.12 (0.07, 0.16) | 0.11 (0.07, 0.16) | 0.10 (0.05, 0.14) | *0.04* |
| **Hazardous alcohol use** |  |  |  |  |  |
| Educational level | 0.07 (0.03, 0.11) | 0.02 (-0.02, 0.05) | -0.01 (-0.04, 0.03) | -0.03 (-0.07, 0.01) | *<0.01* |
| Household income | 0.01 (-0.03, 0.06) | -0.03 (-0.07, 0.01) | -0.04 (-0.08, 0.00) | -0.06 (-0.10, -0.02) | *<0.01* |
| Health insurance subsidy | -0.01 (-0.07, 0.04) | -0.06 (-0.11, -0.01) | -0.09 (-0.14, -0.04) | -0.11 (-0.16, -0.06) | *<0.01* |
| **Diabetes** |  |  |  |  |  |
| Educational level | 0.07 (0.05, 0.10) | 0.05 (0.03, 0.07) | 0.06 (0.04, 0.08) | 0.06 (0.03, 0.08) | *0.35* |
| Household income | 0.11 (0.08, 0.13) | 0.07 (0.04, 0.09) | 0.09 (0.06, 0.11) | 0.07 (0.05, 0.10) | *0.12* |
| Health insurance subsidy | 0.11 (0.07, 0.15) | 0.08 (0.04, 0.11) | 0.09 (0.05, 0.12) | 0.08 (0.05, 0.11) | *0.07* |
| **Smoking** |  |  |  |  |  |
| Educational level | 0.20 (0.16, 0.23) | 0.22 (0.18, 0.26) | 0.22 (0.18, 0.25) | 0.19 (0.15, 0.22) | *0.53* |
| Household income | 0.22 (0.18, 0.26) | 0.22 (0.18, 0.26) | 0.23 (0.19, 0.27) | 0.18 (0.14, 0.22) | *0.17* |
| Health insurance subsidy | 0.20 (0.14, 0.25) | 0.21 (0.16, 0.26) | 0.20 (0.14, 0.25) | 0.16 (0.11, 0.21) | *0.06* |
| **Sedentarity** |  |  |  |  |  |
| Educational level | 0.16 (0.12, 0.19) | 0.14 (0.10, 0.17) | 0.17 (0.13, 0.20) | 0.09 (0.06, 0.13) | *0.01* |
| Household income | 0.13 (0.09, 0.17) | 0.10 (0.06, 0.13) | 0.15 (0.11, 0.19) | 0.07 (0.04, 0.11) | *0.07* |
| Health insurance subsidy | 0.01 (-0.03, 0.06) | 0.00 (-0.04, 0.04) | 0.03 (-0.01, 0.08) | -0.06 (-0.10, -0.01) | *<0.01* |

Values are SII (Slope Index of Inequality) with 95% confidence intervals from linear regression models with robust standard errors, adjusted for age, sex, and survey year. CHF: Swiss Francs (1 CHF = 1.10 USD on 11 November 2023). SII = 0.00 indicates no difference in prevalence between most disadvantaged and most privileged socioeconomic groups.

^a^ P-value for linear trend from interaction term between the SII and survey period.

**Figure S1.** Participation rate across the 2008–2019 period, Bus Santé


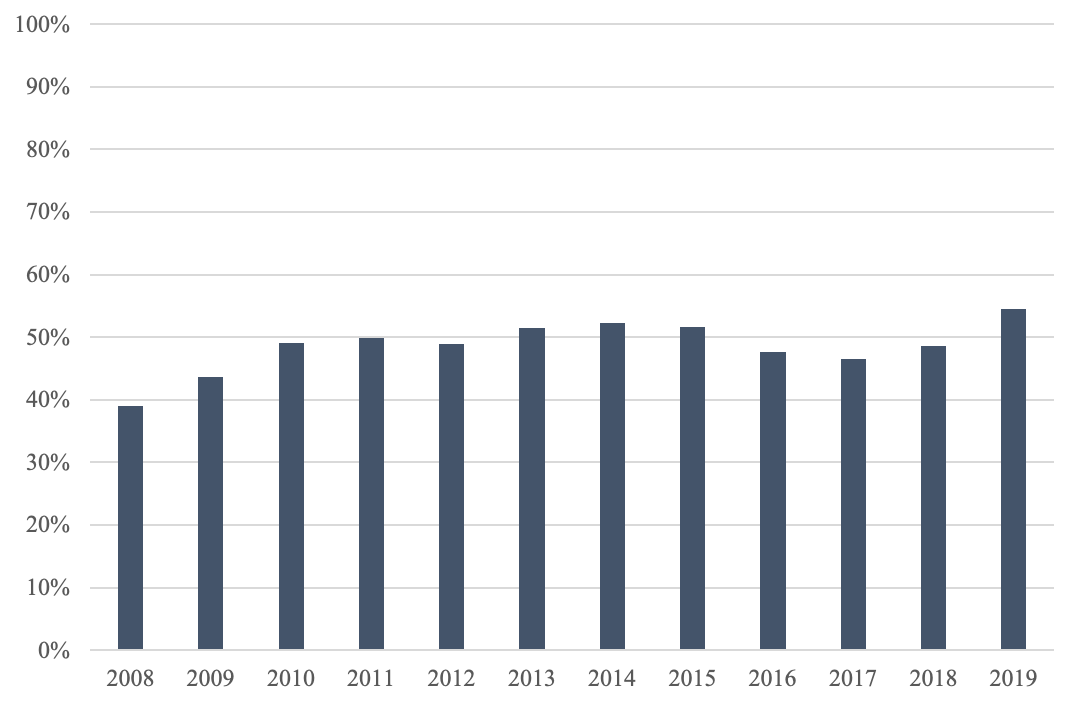

Supplement: Supplementary Data 1 [file mmc1.docx]
